# Supplementary material for: From sequence to enzyme mechanism using multi-label machine learning
Source: BMC Bioinformatics. 2014 May 19;15:150. doi: 10.1186/1471-2105-15-150 (PMC4229970; doi:10.1186/1471-2105-15-150)
Supplement: Additional file 2 — Java code of ml2db. Additional file ml2db_code.tar.gz contains the Java source code to run the multi-label machine learning experiments and save the results to database. The code’s Javadoc is included. [file 1471-2105-15-150-S2.zip › additional file 2/ml2db/ml2db/doc/main/ml2db/weka/DataSetManager.html]

DataSetManager


JavaScript is disabled on your browser.


- Overview
- Package
- Class
- Use
- Tree
- Deprecated
- Index
- Help

- Prev Class
- Next Class

- Frames
- No Frames

- All Classes

- Summary:
- Nested |
- Field |
- Constr |
- Method

- Detail:
- Field |
- Constr |
- Method


main.ml2db.weka

## Class DataSetManager

- java.lang.Object
- - uk.ac.standrews.utils.main.database.Manager
  - - main.ml2db.weka.DataSetManager

- Direct Known Subclasses:
  :   MulanDataSetManager

  ---

    

  ```
  public class DataSetManager
  extends uk.ac.standrews.utils.main.database.Manager
  ```

  DataSet generates a collection of Weka instances (rows of the ARFF file),
  their attributes (column names) and their attribute values (cell values) The
  last attribute is the class attribute.

  Version:
  :   20 Feb 2008

  Author:
  :   Luna De Ferrari ldf(at)st-andrews.ac.uk

- - ### Field Summary

    Fields

    | Modifier and Type | Field and Description |
    | --- | --- |
    | `static java.lang.String` | `DUMMY_VALUE` |
  - ### Constructor Summary

    Constructors

    Constructor and Description

    `DataSetManager(Arff generator)`
  - ### Method Summary

    Methods

    | Modifier and Type | Method and Description |
    | --- | --- |
    | `void` | `fillArff()` |
    | `Arff` | `getArffGenerator()` |
    | `ArffPropsFromFile` | `getArffProperties()` |
    | `java.lang.String` | `getClassName()` |
    | `DataSetDbLoader` | `getDataLoader()` |
    | `DataSetChecker` | `getDataSetChecker()` |
    | `DataSetGenerator` | `getDataSetGenerator()` |
    | `java.lang.String` | `getDataSetName()` |
    | `DataSetWriter` | `getDataSetWriter()` |
    | `boolean` | `initialisationIsCorrect()` |
    | `void` | `writeBaseArff()` |

    - ### Methods inherited from class java.lang.Object

      `equals, getClass, hashCode, notify, notifyAll, toString, wait, wait, wait`

- - ### Field Detail


    - #### DUMMY\_VALUE

      ```
      public static final java.lang.String DUMMY_VALUE
      ```

      See Also:
      :   Constant Field Values
  - ### Constructor Detail


    - #### DataSetManager

      ```
      public DataSetManager(Arff generator)
      ```

      Parameters:
      :   `generator` - a generator object to write this dataset to file
  - ### Method Detail


    - #### fillArff

      ```
      public void fillArff()
      ```


    - #### getArffGenerator

      ```
      public Arff getArffGenerator()
      ```


    - #### getArffProperties

      ```
      public ArffPropsFromFile getArffProperties()
      ```


    - #### getClassName

      ```
      public java.lang.String getClassName()
      ```


    - #### getDataLoader

      ```
      public DataSetDbLoader getDataLoader()
      ```


    - #### getDataSetChecker

      ```
      public DataSetChecker getDataSetChecker()
      ```


    - #### getDataSetGenerator

      ```
      public DataSetGenerator getDataSetGenerator()
      ```


    - #### getDataSetName

      ```
      public java.lang.String getDataSetName()
      ```


    - #### getDataSetWriter

      ```
      public DataSetWriter getDataSetWriter()
      ```


    - #### initialisationIsCorrect

      ```
      public boolean initialisationIsCorrect()
      ```


    - #### writeBaseArff

      ```
      public void writeBaseArff()
      ```


- Overview
- Package
- Class
- Use
- Tree
- Deprecated
- Index
- Help

- Prev Class
- Next Class

- Frames
- No Frames

- All Classes

- Summary:
- Nested |
- Field |
- Constr |
- Method

- Detail:
- Field |
- Constr |
- Method
